# DYADIC EFFECTS ON DEPRESSIVE SYMPTOMS OF SPOUSE CAREGIVERS AND THEIR CARE RECIPIENTS: EVIDENCE FROM CHINA

**DOI:** 10.1093/geroni/igac059.662

**Published:** 2022-12-20

**Authors:** Jinyu Liu, Yifan Lou, Lydia Li, Hongwei Xu, Zhenmei Zhang

**Affiliations:** Columbia University, New York, New York, United States; Columbia University, New York, New York, United States; University of Michigan, Ann Arbor, Michigan, United States; Queens College in the City University of New York, Queens, New York, United States; Michigan State University, East Lansing, Michigan, United States

## Abstract

**Objectives:**

The likelihood of providing care to a spouse in middle and older ages has increased as life expectancy increases, but knowledge about how the caregiver and care recipient influence each other’s mental health is limited. This study examined whether a partner’s physical, cognitive, and mental health in a spousal caregiving dyad are associated with the other partner’s depressive symptoms in China and whether the dyadic effects vary by gender.

**Methods:**

This study used data from Wave 3 (2015) and Wave 4 (2018) follow-up surveys of the China Health and Retirement Longitudinal Study (CHARLS). The analytic sample featured 1,245 dyads of care recipients aged 45 or older and their spouse caregivers. The Actor-Partner Interdependence Model was used to test the dyadic effects among all couples in the analytic sample, couples with wife caregivers and couples with husband caregivers, respectively.

**Results:**

We found that caregiver’s depressive symptoms at Wave 3 were significantly associated with care recipient’s depressive symptoms at Wave 4 in the full sample. Regardless of caregiver or care recipient roles, wives’ mental health was impacted by their husbands’ depressive symptoms, but not vice versa. Wife recipient’s cognitive impairment was associated with husband caregiver’s lower depressive symptoms. Discussion: This study sheds light on the mental health of couples in the context of caregiving in China. The findings indicate that interventions to support couples in a caregiving dyad need to consider the influence they have on each other, and the gender and health conditions of each in the dyad.

